# Cross feeding of glucose metabolism byproducts of *Escherichia coli* human gut isolates and probiotic strains affect survival of *Vibrio cholerae*

**DOI:** 10.1186/s13099-016-0153-x

**Published:** 2017-01-17

**Authors:** Chirantana Sengupta, Manjula Ekka, Saurabh Arora, Prashant D. Dhaware, Rukhsana Chowdhury, Saumya Raychaudhuri

**Affiliations:** 1 CSIR-Institute of Microbial Technology, Sector 39A, Chandigarh, 160036 India; 2 CSIR-Indian Institute of Chemical Biology, 4 Raja SC Mullick Road, Kolkata, 700032 India

**Keywords:** *V. cholerae* survival, Cocultures, *E. coli* Nissle 1917, *E. coli* glucose transport mutants

## Abstract

*Vibrio cholerae* converts glucose into either acid or the neutral end product acetoin and its survival in carbohydrate enriched media is linked to the nature of the byproducts produced. It has been demonstrated in this study that *Escherichia coli* strain isolated from the gut of healthy human volunteers and the commonly used probiotic *E. coli* Nissle strain that metabolize glucose to acidic byproducts drastically reduce the survival of *V. cholerae* strains irrespective of their glucose sensitivity and acetoin production status. Accordingly, *E. coli* glucose transport mutants that produce lower amounts of acidic metabolites had little effect on the survival of *V. cholerae* in cocultures. Thus, cross feeding of byproducts of glucose metabolism by heterologous bacteria modulates the survival of *V. cholerae* in glucose rich medium suggesting that composition of the gut microbiota could influence the outcome of *V. cholerae* infection especially when glucose based ORS is administered.

**Electronic supplementary material:**

The online version of this article (doi:10.1186/s13099-016-0153-x) contains supplementary material, which is available to authorized users.

## Background

Successful infection by bacterial pathogens depends primarily upon a complex interplay between bacterial virulence factors and host responses and defense systems. Recently however, it is becoming apparent that presence of other microorganisms might profoundly influence the outcome of bacterial infections including that of *Vibrio cholerae* [1]. In this study we demonstrate that presence of heterologous organisms, with ability to produce acidic byproducts of glucose metabolism might modulate the survival of *V. cholerae* under glucose rich conditions.

All recorded cholera pandemics have been caused by strains of the O1 serogroup of *V. cholerae* that can be classified into two major biotypes, classical and El Tor. Although the classical and El Tor biotypes are closely related, several biochemical and genetic differences have been reported between the two biotypes including a unique difference in carbohydrate metabolism. In the presence of exogenous sugars the classical biotype strains (e.g. O395) produced organic acids resulting in a sharp decrease in media pH and drastic loss of viability. El Tor strains (e.g. N16961) have evolved to metabolize sugars to produce acetoin, a neutral fermentation end-product that did not inhibit bacterial growth [2]. It has been suggested that the ability to metabolize sugars without production of growth inhibitory acidic products might account for the evolutionary fitness of the *V. cholerae* El Tor biotype by virtue of which it displaced the classical biotype as a cause of epidemic cholera [2].

Here, we postulate that cross feeding of byproducts of glucose metabolism by heterologous bacteria might modulate the survival of *V. cholerae* in glucose rich growth medium. As proof of concept, *V. cholerae* classical and El Tor biotype strains were co-cultured in the presence of glucose with *E.coli* strains that produce acidic byproducts of glucose metabolism, and the effect of co-culturing on *V. cholerae* survival was determined. We observed a drastic loss of viability of *V. cholerae* strains irrespective of their acetoin production status in the co-culture with *E. coli* strains under carbohydrate rich condition. On the other hand, *E. coli* glucose transport mutants that produce lower amounts of acidic metabolites had little effect on the survival of *V. cholerae* in co-cultures.

## Methods

### Strains

All bacterial strains used for this study have been described in Additional file 1: Table S1. Bacterial cultures grown to the logarithmic phase in Luria broth (LB) were stored in glycerol (10% v/v) at −80 °C. When required, streptomycin was used at a concentration of 100 μg/ml.

### Coculture studies

*V. cholerae* El Tor strain N16961 (Sm^R^) or classical strain O395 (Sm^R^) and *E. coli* strains mentioned in Table S1, were grown in LB medium up to mid-logarithmic phase. Cocultures (1:1) were set up in LB or LB containing 1% glucose (LBG). Monocultures were set up in LB and LBG in a similar manner as a control. At regular intervals aliquots of the cultures were removed and the number of *V. cholerae* cells was enumerated by serial dilution and plating on Luria agar containing streptomycin (100 μg/ml). At all time points, pH in the culture supernatants was measured. Statistical significance of the data has been calculated and expressed as ±SD in all experiments.

### Spotting assays

Survival of *V. cholerae* N16961 strain in cell free conditioned medium was assayed by spotting on LA plates containing streptomycin (100 μg/ml). Cell free conditioned medium was prepared from 12 h cultures of N16961 and *E. coli* strains grown in LB or LBG at 37 °C with aeration. Mid-logarithmic phase *V. cholerae* N16961 was resuspended in the conditioned medium and cultures were allowed to grow for 12 h at 37 °C with aeration. Survival was assayed by spotting dilutions (10^−3^–10^−6^) on LB agar plates containing streptomycin to select *V. cholerae* N16961 (Sm^R^).

### Results and discussion

To ascertain how the survival of *V. cholerae* is influenced by heterologous bacteria in glucose enriched medium, *V. cholerae* classical and El Tor biotypes strains were co-cultured with wild type *E. coli* strains (Additional file 1: Table S1) in the presence or absence of glucose. The *E. coli* strains produce largely acidic metabolites upon glucose fermentation [3–5]; it is therefore conceivable that the survival of *V. cholerae* may be affected in the co-culture with *E. coli* in LBG.

**Fig. 1:**
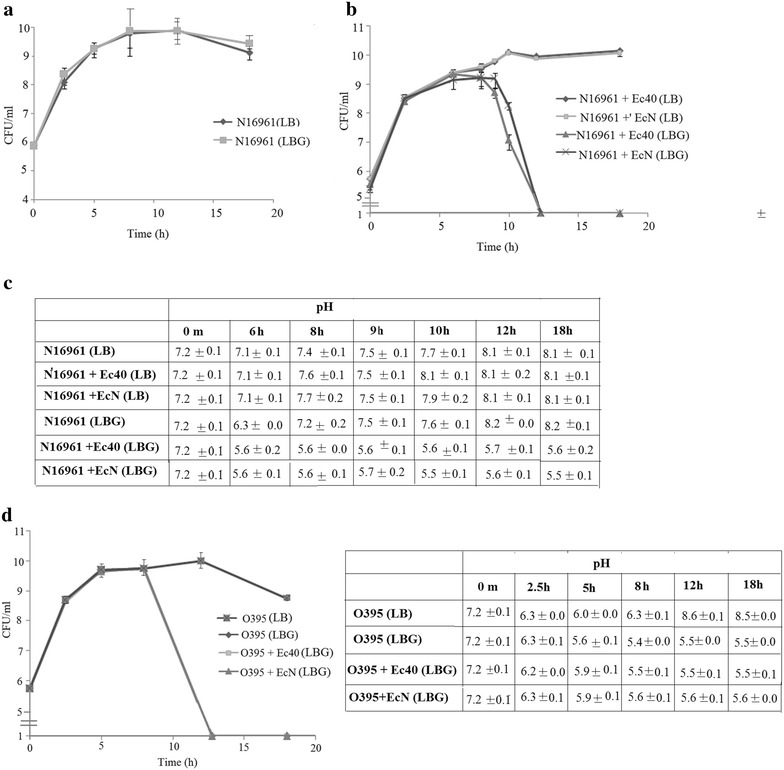
Cocultures of *V. cholerae* with *E. coli* strains.CFU of *V. cholerae* El Tor strain N16961 (Sm^R^) was enumerated in individual cultures (**a**) or in cocultures (1:1) with *E. coli* 40 (Ec40) or *E. coli* Nissle (EcN) (**b**) at different time points. At all time points, pH of the culture supernatants was measured (**c**). CFU of *V. cholerae* classical strain O395 (Sm^R^) in mono- and co-cultures was similarly assayed and pH of the culture supernatants was measured (**d**)

**Fig. 2:**
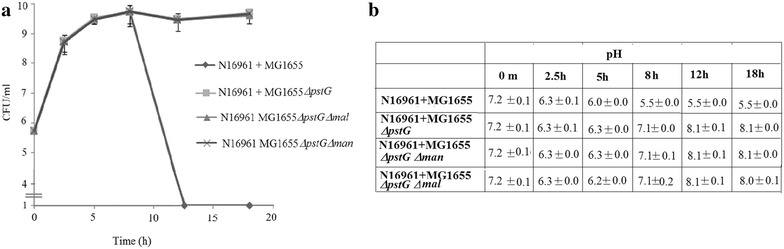
Cocultures of *V. cholerae* with *E. coli* WT and glucose transport mutants. *V. cholerae* N16961 (Sm^R^) was grown together with *E. coli* MG1655 or glucose transport mutants (1:1 ratio) in LB medium containing glucose (1%). At regular intervals CFU of *V. cholerae* cells was determined (**a**) and pH of the culture supernatants was measured (**b**)

As reported earlier, *V. cholerae* classical biotype strain O395 exhibited severe growth defect in Luria broth medium supplemented with 1% glucose (LBG) since this biotype produces acidic byproducts of glucose metabolism. On the other hand, *V. cholerae* El Tor biotype strain N16961 that metabolizes glucose to acetoin, could grow normally in LBG [2]. However, when the strains were grown in co-cultures with *E. coli* 40 or *E. coli* Nissle, a drastic decline in cell count of both the *V. cholerae* strains was observed concomitant with acidification of the growth media presumably due to acidic glucose fermentation metabolites produced by *E. coli* (Fig. 1). Interestingly, it was noted that although N16961 is capable of producing acetoin in LBG medium, it failed to overcome the lethal effect of strong acidification caused by the *E. coli* strains. These results suggest that *E. coli* strains by virtue of their ability to metabolize glucose to acidic byproducts and cause acidification of the growth medium in LBG, affect survival of *V. cholerae* strains irrespective of the acetoin producing capability of the latter, when the *E. coli* and *V. cholerae* strains are grown together. To examine if acidification of the medium by acidic byproducts of glucose metabolism during growth of *E. coli* cultures in LBG was primarily responsible for the killing of *V. cholerae* strains in the co-cultures, the *V. cholerae* strains were next co-cultured with the *E. coli* MG1655 glucose transport mutants (Additional file 1: Table S1). In these co-cultures much lower acidification of the growth medium was observed and survival of the *V. cholerae* strains was much higher than that in co-cultures with the wild type *E. coli* strains (Fig. 2) strongly suggesting that glucose uptake and metabolism produced acidic byproducts and the resulting acidification of the media in wild type *E. coli* cultures resulted in severe decline in cell count of *V. cholerae* strains in LBG. Suspension of *V. cholerae* strain N16961 in conditioned medium prepared from 12 h cultures of *E. coli* resulted in loss of cell count indicating that cell–cell contact was not necessary for the decline in cell count of *V. cholerae* in the presence of the *E. coli* strains (Additional file 2: Fig. S1).

Based on its interaction with host, *E. coli* strains can be categorized broadly into non pathogenic commensal and pathogenic groups. The pathogenic groups can be further divided into intestinal pathogenic and extra intestinal pathogenic strains [6, 7]. Interestingly, the commensal and probiotic *E. coli* EcN strain has been exploited clinically to ameliorate the burden of ulcerative colitis and Crohn’s disease [8–10]. Other than clinical use, EcN as well as other non pathogenic *E. coli* strains have been evaluated for their potential as delivery vehicles [11], in reducing intestinal colonization of *Salmonella typhimurium* [12] and in cancer therapy [13]. Though non pathogenic commensal and probiotic *E. coli* strains have tremendous potential for therapeutic use, it must be borne in mind that efficacy of such strains as probiotics is strongly dependent on the composition of intestinal microbiota and immune status of host [14].

## Conclusion

In essence, this study suggests that preponderance of bacterial strains that metabolize glucose to acidic compounds in the gut might hinder *V. cholerae* survival. The result is particularly important as glucose based oral rehydration therapy is currently highly recommended during cholera infections. Thus, selected *E. coli* strains isolated from healthy human gut and commonly used probiotic *E. coli* Nissle that metabolize glucose to acidic byproducts could be used as probiotics together with ORT for the control of cholera.

## Additional files

**Additional file 1: Table S1.** List of strains.

**Additional file 2: Figure S1**. Survival of *V. cholerae* N16961 strain in cell free conditioned medium prepared from 12 h cultures of N16961 and *E. coli* strains grown in LB (**A**) or LBG (**B**). Survival was assayed by spotting dilutions (10^−3^ to 10^−6^) on LB agar plates containing streptomycin (100 μg/ml) different to select *V. cholerae* N16961 (Sm^R^).
