# Supplementary material for: Cross feeding of glucose metabolism byproducts of Escherichia coli human gut isolates and probiotic strains affect survival of Vibrio cholerae
Source: Gut Pathog. 2017 Jan 17;9:3. doi: 10.1186/s13099-016-0153-x (PMC5240293; doi:10.1186/s13099-016-0153-x)
Supplement: Supplementary file 1 — Additional file 1: Table S1. List of strains. [file 13099_2016_153_MOESM1_ESM.doc]

| Strains | Description | Acetoin status | Source/reference |
| --- | --- | --- | --- |
| ***Vibrio cholerae*** **stains** |  |  |  |
| N16961 | El Tor, Ogawa, Sm^r^ | +++ | [16] |
| O395 | Clasical, Ogawa or Inaba, Sm^r^ | --- | Laboratory collection |
| ***Escherichia coli*** **strains** |  |  |  |
| EcN | *E. coli* Nissle 1917 human isolates, probiotic strain | --- | Prof. Rudolf von Bünau, Ardeypharm |
| Sample 40 | *E.coli* sample 40 human isolates, “A” phylotype | --- | [15] |
| Sample 89 | *E. coli* sample 89 human isolates “A” phylotype | --- | [15] |
| MG1655 | *E.coli* K-12 | --- | Prof. Katja Bettenbrock, MPI Magdeburg, Germany |
| MG1655Δ*ptsG* | sugar transport mutants | --- | Prof. Katja Bettenbrock, MPI Magdeburg, Germany |
| MG1655 Δ*ptsG*Δ*malEFG* | sugar transport mutants | --- | Prof. Katja Bettenbrock, MPI Magdeburg, Germany |
| MG1655 Δ*ptsG*Δ*manXYZ* | sugar transport mutants | --- | Prof. Katja Bettenbrock, MPI Magdeburg, Germany |

Table S1: List of strains
