# Supplementary figures and images for: Cross feeding of glucose metabolism byproducts of Escherichia coli human gut isolates and probiotic strains affect survival of Vibrio cholerae
Source: Gut Pathog. 2017 Jan 17;9:3. doi: 10.1186/s13099-016-0153-x (PMC5240293; doi:10.1186/s13099-016-0153-x)

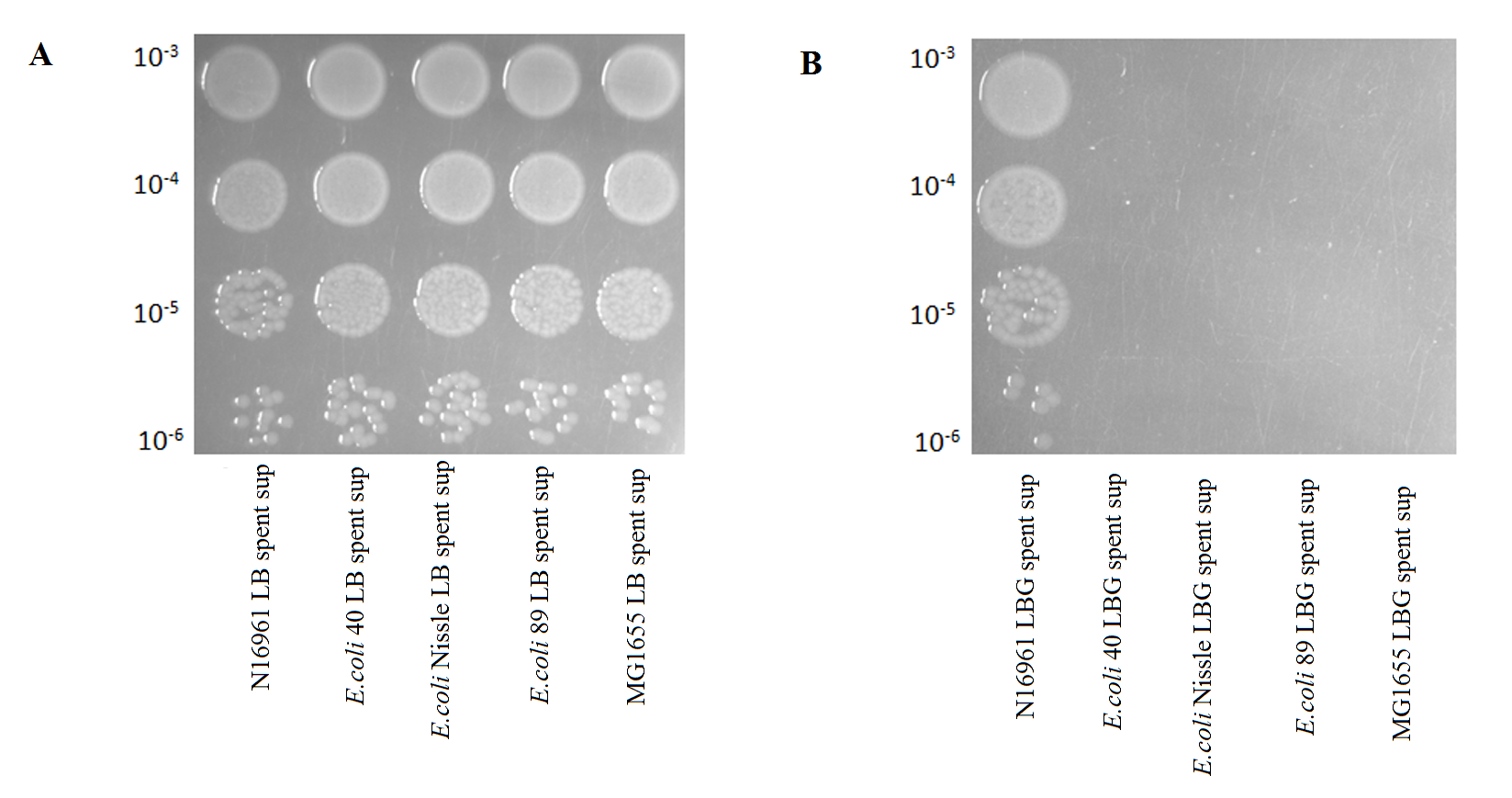

Supplement: Supplementary file 2 — Additional file 2: Figure S1. Survival of V. cholerae N16961 strain in cell free conditioned medium prepared from 12 h cultures of N16961 and E. coli strains grown in LB (A) or LBG (B). Survival was assayed by spotting dilutions (10−3 to 10−6) on LB agar plates containing streptomycin (100 μg/ml) different to select V. cholerae N16961 (SmR). [file 13099_2016_153_MOESM2_ESM.tif]
